# Observed physical and biogeochemical variability due to tropical cyclone Mocha using glider observations in the Bay of Bengal

**DOI:** 10.1038/s41598-026-43528-2

**Published:** 2026-04-21

**Authors:** V. P. Thangaprakash, N. Sureshkumar, K. Siva Srinivas, A. Chandramouli, Sai Theagarajan, Y. Rajasekhar, Virendra Kumar, M. Ramesh Kumar

**Affiliations:** Indian National Centre for Ocean Information Services (INCOIS), Ministry of Earth Sciences, Govt. of India, Hyderabad, 500090 India

**Keywords:** Physical and biogeochemical variables, Tropical Cyclones, Deep-Sea Gliders, Climate sciences, Ecology, Ecology, Environmental sciences, Ocean sciences, Plant sciences

## Abstract

Deep-sea glider measurements of temperature, salinity, chlorophyll-a, and dissolved oxygen (DO) collected during Tropical Cyclone (TC) *Mocha* (09-15 May 2023) provided a unique opportunity to examine the response of physical and biogeochemical parameters in the Bay of Bengal during the secondary TC peak season. Glider measurements show a significant reduction in SST (~2.5°C) and a mild increase in SSS (~0.9 psu) in response to the TC. Similarly,biogeochemical observations show a strong response, with increase in near-surface chlorophyll concentration (1.6 mg m^-3^) and DO (10 μM) driven by TC-induced vertical mixing and upwelling. The second near-surface chlorophyll peak (1.7 mg m^-3^), observed eight days after the passage of TC, was substantially stronger than the first peak (0.8 mg m^-3^) that occurred immediately after the TC passed. This difference may be attributed to extensive cloud cover during the first chlorophyll peak, which likely limited light availability at the surface. Both chlorophyll and dissolved oxygen reached their maximum concentrations approximately 8–9 days after the TC passed the study location. Statistical analysis indicates enhanced near-surface diurnal variability in the DO during the post-TC bloom period, compared to the non-bloom period. Further, the model shows a weaker response to the TC than that observed in the glider measurements.

**Supplementary Information:**

The online version contains supplementary material available at 10.1038/s41598-026-43528-2.

## Introduction

Tropical Cyclones (TCs) can cause severe damage to life and property, which ultimately have socio-economic impacts on the country. The Bay of Bengal (BoB) is highly susceptible to tropical cyclone (TC) formation, with an average of 3–4 TCs occurring annually^1^. The TCs in the BoB occur particularly during the pre-monsoon (May-June: secondary) and post-monsoon (October-December: primary) seasons and have a profound impact on the surrounding regions^2,3^.

The BoB is known for its unique salinity stratification at the near-surface, resulting from the freshwater received from seasonal monsoon rainfall and river discharge, which leads to a shallow mixed layer (ML) and stabilizes the ocean, affecting the transfer of energy from the ocean to the atmosphere^4–8^. This strong haline stratification also leads to low biological productivity in the BoB by inhibiting the upward flux of nutrient-rich subsurface waters into the euphotic zone, and plays a significant role in the seasonal evolution of phytoplankton variability in the BoB^9–14^. The monsoon cloud cover and river suspended sediments transported into the BoB can cause a reduction in the availability of energy in the upper water column^9,15,16,14^.

Earlier studies have shown that the seasonal and interannual variability of chlorophyll is influenced by various physical processes, including open-ocean upwelling, eddies, westward-propagating upwelling Rossby waves, and vertical mixing caused by wind events^14,17,18^. These processes can erode the near-surface stratification and lead to the enhancement of chlorophyll in the near-surface layer through the upward flux of nutrient-rich subsurface water into the euphotic zone^14,17–23^.

Furthermore, various studies have documented the enhancement of chlorophyll in response to TCs in the BoB^18,24–30^. The drawbacks of most of these earlier studies include a lack of realistic information during TCs due to data gaps from satellite-based estimation, which are often hindered by large cloud cover, and the infeasibility of obtaining ship-borne in-situ observations during violent wind and high wave conditions associated with TCs. Although a few earlier studies reported the impact of TCs on physical and biogeochemical variability using autonomous profiling biogeochemical (BGC) Argo floats, they are limited by temporal and vertical resolution, as Argo floats measure at predefined depths^31–34^. In addition, Argo floats obtain continuous measurements for nearly 3–5 years (depending on battery and environmental conditions) without calibration, which can lead to drift in sensor measurements^34^. Thus, autonomous underwater vehicles (AUVs) such as deep-sea gliders (SGs) can collect high-quality upper-ocean measurements with high vertical and temporal resolution. These vehicles, which can be remotely configured via satellite communication at any time from shore, play a crucial role, especially during tropical cyclones (TCs). Thus, a better understanding of the upper ocean’s physical and biogeochemical parameters in response to TC using a deep-sea glider is worth investigating and is crucial to simulate these parameters in the coupled physical and biogeochemical models to have better forecasts.

**Fig. 1:**
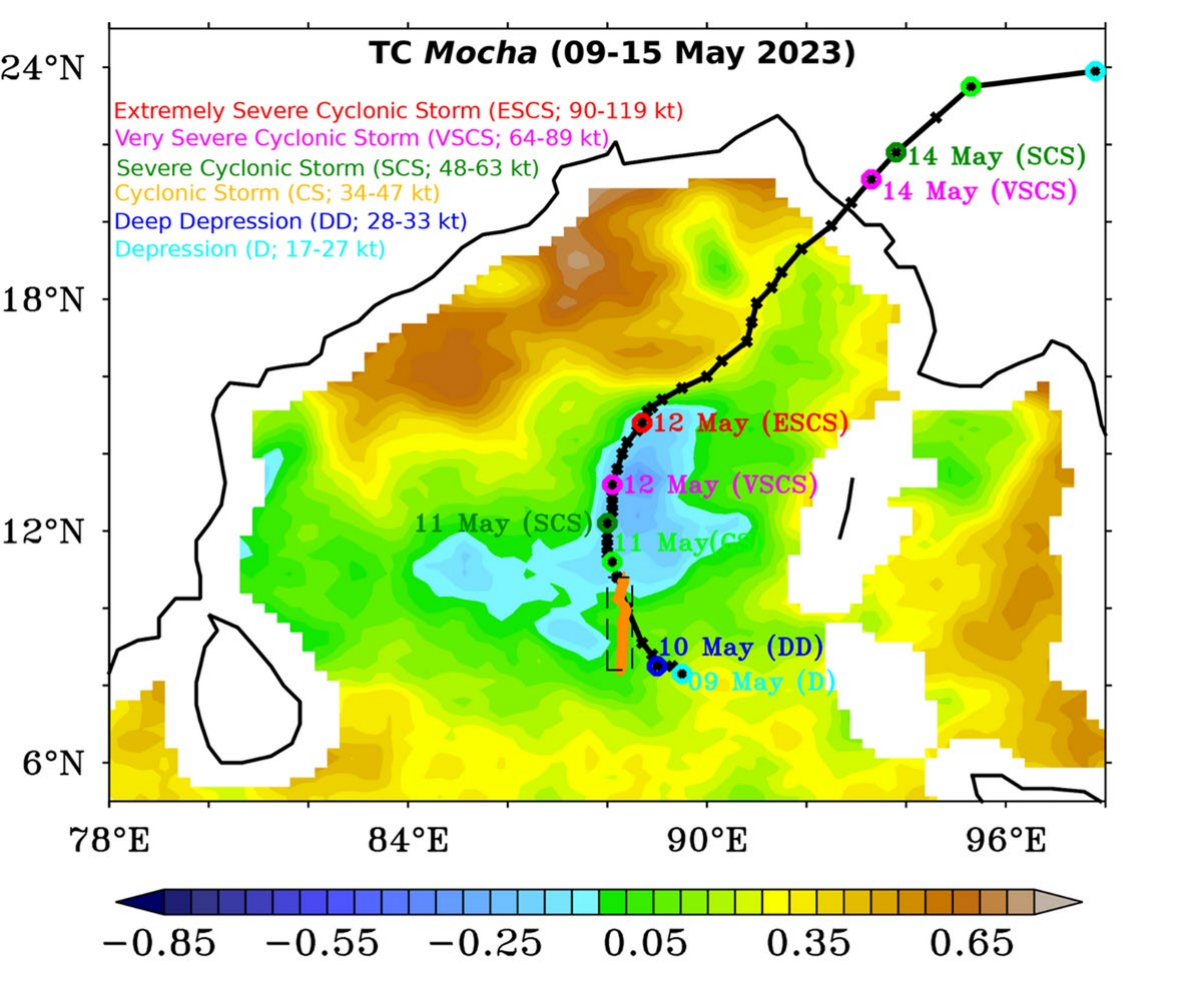
The monthly SST anomaly (May 2023) from MW-OI SST was overlaid with six-hourly best track (black line) and intensity (open colored circles corresponding to the dates and intensity) of TC *Mocha* obtained from the Regional Specialised Meteorological Centre (RSMC), IMD, India (IMD Best track data*).* The classification of tropical systems by IMD with respect to wind speed is classified as Depression (D) (17–27 knot; kt), Deep Depression (DD) (28–33 kt), Cyclonic Storm (CS) (34–47 kt), Severe Cyclonic Storm (SCS) (48–63 kt), Very Severe Cyclonic Storm (VSCS) (64–89 kt), and Extremely Severe Cyclonic Storm (ESCS) (90–119 kt) and are marked as light blue, blue, green, orange, purple and red circles respectively along the track of TCs. The numbers adjacent to each circle indicate the dates. Orange triangles in the figure indicate the glider location from 05–25 May 2023, and TC *Mocha* crosses the glider location from 10–11 May 2023. The black-dashed box (8.4°N–10.8°N and 88°E–88.5°E) indicates the study location, and marks the glider transect for the pre- and post-TC impact periods. This figure was generated using PyFerret (v7.63).

The Indian National Centre for Ocean Information Services (INCOIS), Ministry of Earth Sciences (MoES), Government of India, has deployed deep-sea gliders (SG’s) to monitor and understand the variability of deep ocean physical and biogeochemical parameters in the northern Indian Ocean as part of Deep Ocean Mission (DOM) under Development of Ocean Climate Change Advisory Services (OCCAS). While performing the meridional transects from 17.5^°^N to 5^°^S along 88.47^°^E in the BoB, the glider has crossed the track of the TC *Mocha* (09–15 May 2023; Fig. 1) on 10–11 May 2023 and provided high-quality physical and BGC measurements in response to this TC. Initially, the glider was configured to measure only downward profiling upto 1000 m depth with surfacing every 5–6 h. During TC, the glider was triggered to obtain both physical and biogeochemical measurements at very high temporal (profiles at approximately 30–40 min) and vertical resolution (upto 200 m depth at 1 m intervals) from 10 to 12 May 2023 (Fig. 1). Thus, the first glider measurements obtained before and after the passage of the TC *Mocha* in the BoB during the pre-monsoon season give a distinctive advantage and unprecedented opportunity to understand the observed variability of upper ocean physical and biogeochemical response to TC *Mocha*. Moreover, the high-resolution BGC measurements from the glider during both day and nighttime profiles helped us perform quenching corrections to the fluorescence data of the day profiles.

The main objective of this study is to understand,

(i) Role of TC-induced mixing and upwelling on the observed variability of the upper ocean physical and biogeochemical processes.
(ii) Diurnal variability of chlorophyll and dissolved oxygen (DO) measurements from the glider before and after the TC *Mocha*.
(iii) To assess the model’s capability in simulating the physical and biogeochemical responses to a TC *Mocha*, based on glider measurements.

### Results and Discussion

### Dynamic and thermodynamic response to TC *Mocha* passage

**Fig. 2:**
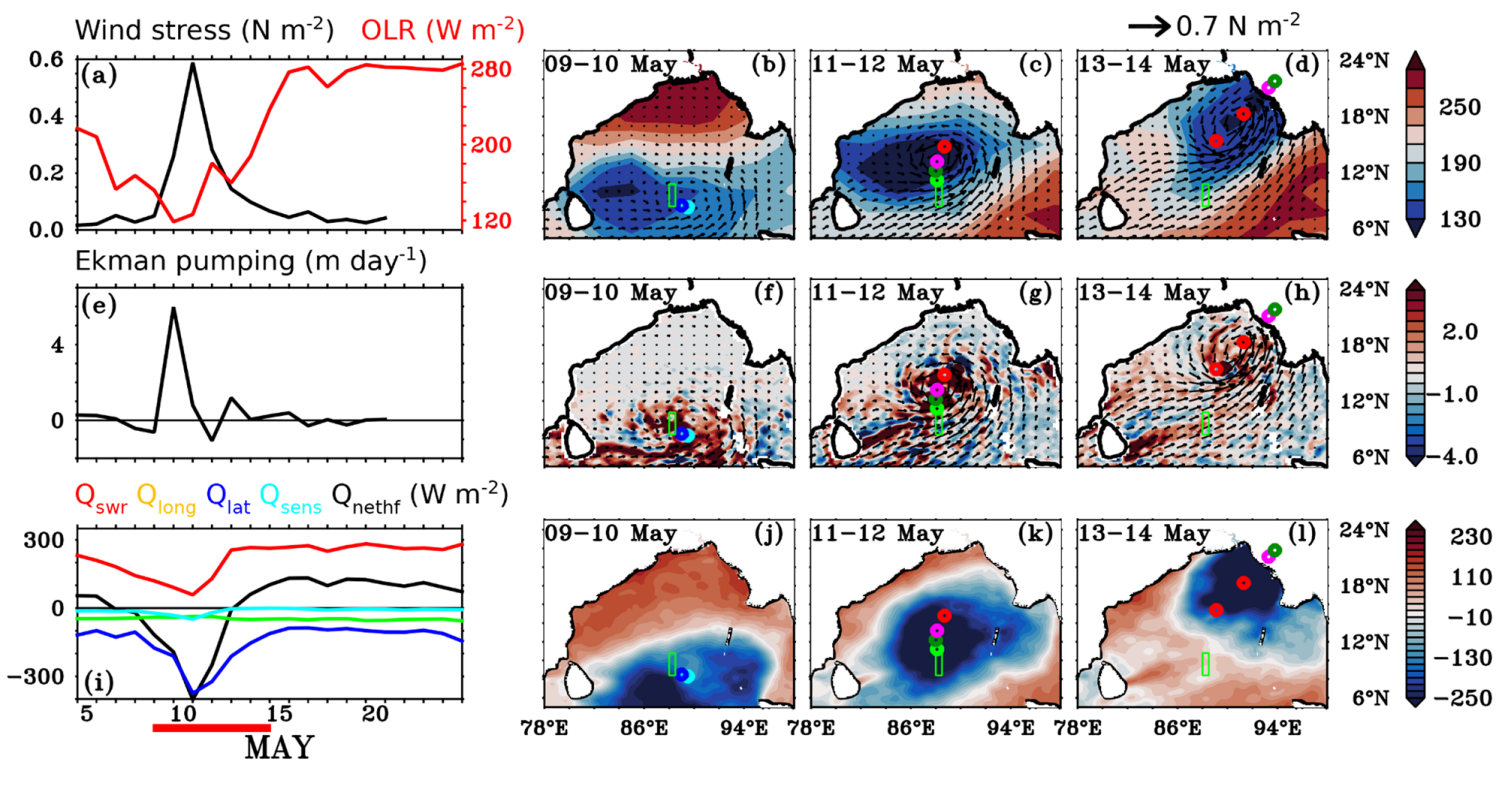
Temporal evolution of (**a**) wind stress (left-hand axis; black line; N m^-2^) from ASCAT and Outgoing Longwave Radiation (OLR; right-hand axis; red line; W m^-2^) from NOAA uninterpolated, (**e**) Ekman pumping velocity (m day^-1^), (**i**) Net shortwave radiation (Q_swr_; red), Net longwave radiation (Q_long_; green), latent heat flux (Q_lat_; blue), sensible heat flux (Q_sens_; light blue) and Net surface heat flux (Q_nethf_; black) from ERA-5 reanalysis during 05–25 May 2023 (all units are in W m^-2^ for panel **i**). All the parameters in the panels (**a**), (**e**), and (**i**) were averaged over the box 8.4°N–10.8°N and 88°E–88.5°E, and thick red line at the bottom of the figure indicates the TC *Mocha* period (09–15 May 2023). The panels (**b**), (**c**), and (**d**) represent the spatial evolution of OLR overlaid with wind stress, and panels (**f**), (**g**), and (**h**) represent the spatial evolution of Ekman pumping overlaid with wind stress, and panels (**j**), (**k**), and (**l**) represent the spatial evolution of Q_nethf_. The green rectangular box indicates the study location, earmarking the glider transect during and after the TC period. This figure was generated using PyFerret (v7.63).

Figure 2 shows the temporal evolution of (a) wind stress (N m^-2^) and Outgoing Longwave Radiation (OLR; W m^-2^), (e) Ekman pumping (m day^-1^), and (i) radiative and turbulent heat fluxes (W m^-2^) averaged over a small domain encompassing glider locations (8.4°N–10.8°N and 88°E–88.5°E). Figure 2 shows the spatial evolution of (b-d) OLR (W m^-2^) overlaid with wind stress, (f-h) Ekman pumping (m day^-1^) overlaid with wind stress, and (j-l) Net surface heat flux (Q_net_; W m^-2^) before, during, and after the period of the TC *Mocha*.

The TC *Mocha* significantly impacts atmospheric dynamics, affecting heat fluxes and overall energy transfer within the system. This leads to intense deep convection, extensive cloud cover and very cold cloud tops, resulting in a substantial reduction in OLR, which dropped to a minimum of 119 W m^-2^ on 10 May 2023 (Fig. 2a; red line). Due to the impact of this TC, the wind stress reached a maximum of upto 0.59 N m^-2^, resulting in the cyclonic-induced Ekman divergence (Fig. 2a-h). The Ekman pumping peaked to 6 m day^-1^ on 10 May 2023, indicating upwelling at the study region (Fig. 2e). As the cyclone strengthened while propagating northwards, the regions of divergence and Ekman pumping also shifted northward (Fig. 2f-h).

In response to TC *Mocha*, net shortwave radiation (Q_swr_) decreased significantly by 58 W m^-2^, with the maximum energy loss occurring through latent heat flux (Q_lat_) at -375 W m^-2^. The losses from net longwave radiation (Q_long_) and sensible heat flux (Q_sens_) were comparatively smaller, at -37 W m^-2^ and  -49 W m^-2^, respectively. This resulted in a maximum cooling of -402 W m^-2^ in the net surface heat flux (Q_net_) on May 11, 2023 (Fig. 2i, j-l). The sharp reduction in net shortwave radiation and the minimal cooling observed in net longwave radiation can be attributed to the extensive cloud cover associated with the TC. Latent heat flux plays a crucial role in TC-induced cooling, as the TC gains energy from the underlying ocean through this process, which serves as a primary energy source for their intensification^35^. However, the sensible heat flux does not contribute significantly to cooling, primarily due to the lower temperature gradient between the sea surface and the atmosphere. This is likely a result of the TC’s strong wind-induced vertical mixing and upwelling, which reduce the temperature gradient and limit the potential for substantial sensible heat flux, leading to a smaller contribution to the overall energy budget. The strong winds associated with the TC enhance evaporation rates and promote convection, which in turn increases latent heat flux. Despite turbulent mixing, the moisture content in the atmosphere above the near-surface layer remains elevated, preserving the humidity gradient and sustaining significant latent heat flux. The wind stress, OLR, radiative, and turbulent heat fluxes recovered immediately after the passage of the TC (15 May 2023).

### Variability of the upper ocean temperature and salinity due to TC *Mocha*

Figure 1 shows the monthly SST anomaly for May 2023 along with the track of the TC *Mocha* and glider transect. The negative SST anomaly (ranging from 0 to -0.25 °C) along the TC track in the southern BoB indicates a cooling of the sea surface compared to the long-term average. This cooling is typically caused by the intense ocean mixing and upwelling associated with the strong winds and turbulence generated by the cyclone. As the cyclone moves across the ocean, it stirs the upper ocean layers, bringing cooler water from below to the surface, which lowers the SST. This cooling can impact the cyclone’s intensity, as lower SSTs reduce the amount of heat and moisture available to fuel the TC’s convection and energy dynamics.

**Fig. 3:**
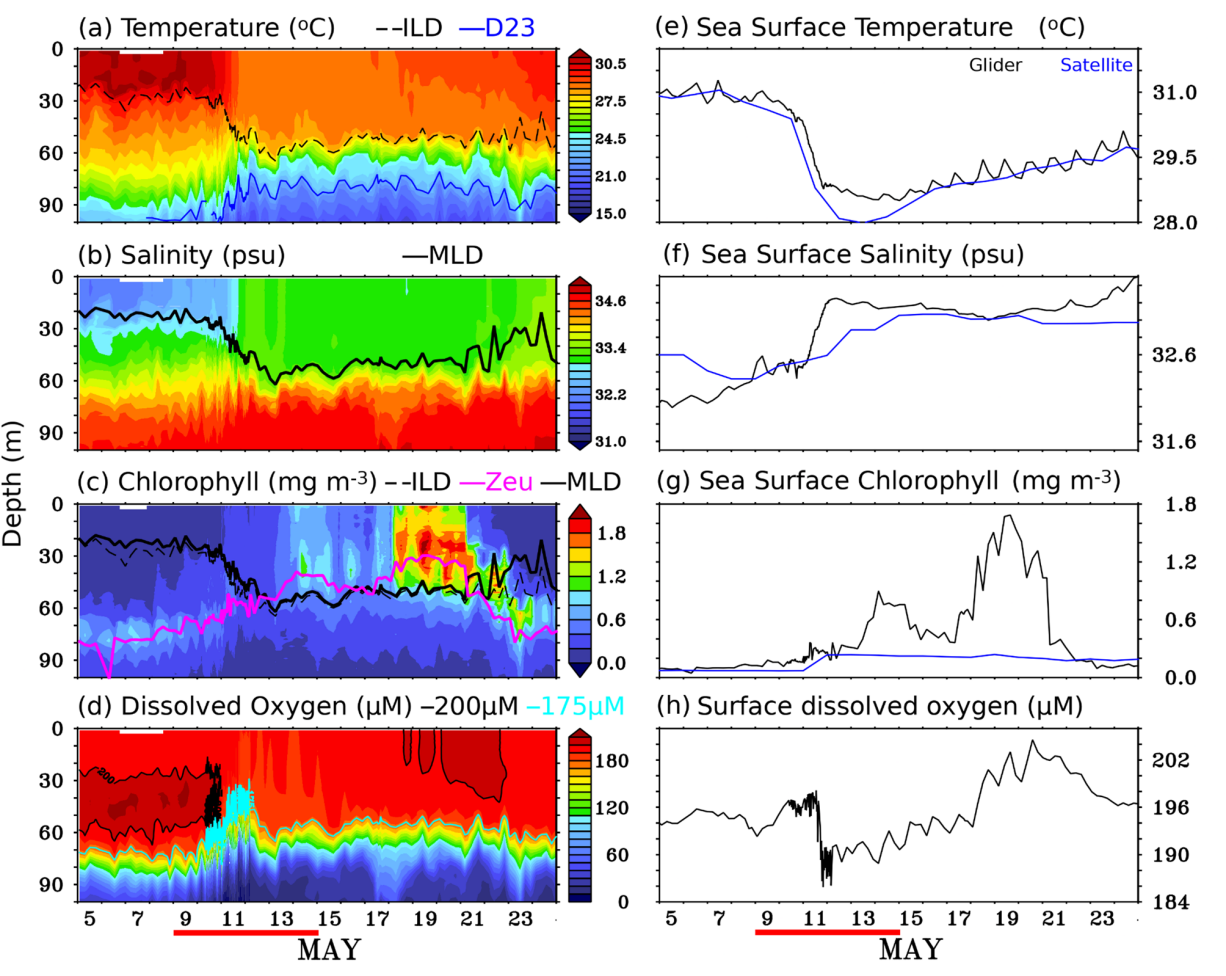
Temporal evolution of (left panel; a-d) depth-time section and sea surface (right panel; e-h) of (a, e) temperature (°C), (b, f) salinity, (c, g) Chlorophyll (mg m^-3^, and (d, h) Dissolved Oxygen (µM) from glider observations during 05–25 May 2023. In panel (**a**), thin dashed black and blue lines are ILD (m) and D23 isotherms; in panel (**b**), a thick black line is MLD (m). In panel (**c**), a thick pink line: euphotic depth, a thick black line is MLD (m), and a thin black dashed line: ILD (m). In panel (**d**), a thin light blue (green) line: depth of 175 (200) µM dissolved oxygen (m). The blue line in panels (e), (**f**), and (**g**), represents data from satellite sources: MW-OI SST, SMAP, and GlobOcean color, and was averaged over the box 8.4°N–10.8°N and 88°E–88.5°E. The thick red line at the bottom of the figure indicates the TC *Mocha* period (09–15 May 2023). This figure was generated using PyFerret (v7.63).

Figure 3 shows the (a-d) time-depth section and (e-h) near-surface physical and biogeochemical variables from the glider measurements during the study period. Before the passage of the TC, the temperature and salinity profile showed typical hydrographic features with warm and low-salinity water in the near-surface layer; cold and high-salinity water in the subsurface layer during May in the southern BoB (sBoB)^4,8,22^ (Fig. 3a and b, and 4a and e). As the TC *Mocha* approached the study area, the glider measurements showed a sudden drop in near-surface temperature starting from 9 May 2023 (Fig. 3a and e, and 4a). In response to the TC, the observed SST from the glider decreased significantly by approximately 2.5 °C, from 31 °C on 09 May 2023 to 28.5 °C on 15 May 2023 (Fig. 3a and e). A similar decline in SST (~ 2.6 °C) was also noticed from satellite data, with a similar magnitude. The satellite SST (averaged over the box of 8.4°N–10.8°N and 88°E–88.5°E) dropped from 30.6 °C on 9 May to 27.98 °C on 13 May 2023 (Fig. 3e). High-temporal measurements from the glider showed diurnal variability in SST before and after the passage of the TC. However, it was not apparent during the TC period, likely due to strong wind-induced vertical mixing and upwelling, as well as extensive cloud cover. After the passage of the TC, near-surface temperatures quickly returned to their pre-TC period. This recovery was clearly visible in the spatio-temporal evolution of the satellite SST, where significant cooling was observed along the TC track, but dissipated within a few days after the cyclone’s passage over the study region (Fig. 5e-h).

**Fig. 4:**
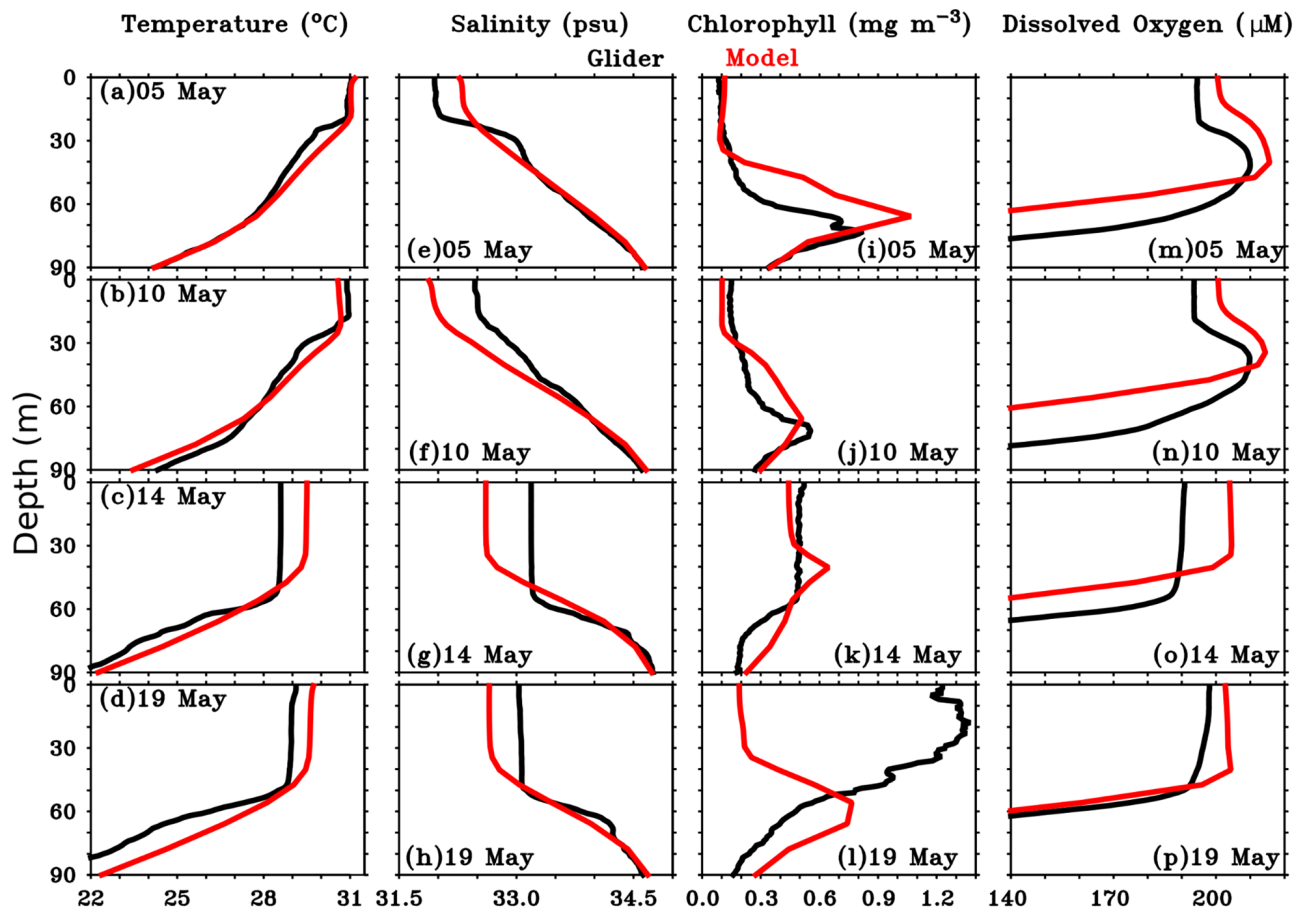
Vertical profiles of (**a**-**d**) Temperature (°C), (**e**-**h**) Salinity (psu), (i-l) Chlorophyll (mg m^-3^, (m-p) Dissolved Oxygen (µM) from glider observation (black line) and model data (red line) before (on 05 May 2023), during (10 May 2023), and after (14 May 2023 and 19 May 2023) the passage of the TC *Mocha* at the study location. The chlorophyll profile shown here (black lines in c, g, k, o) is daily-averaged nighttime profiles from the glider. All the parameters from the model data (red lines) were averaged over the box 8.4°N–10.8°N and 88°E–88.5°E. This figure was generated using PyFerret (v7.63).

**Fig. 5:**
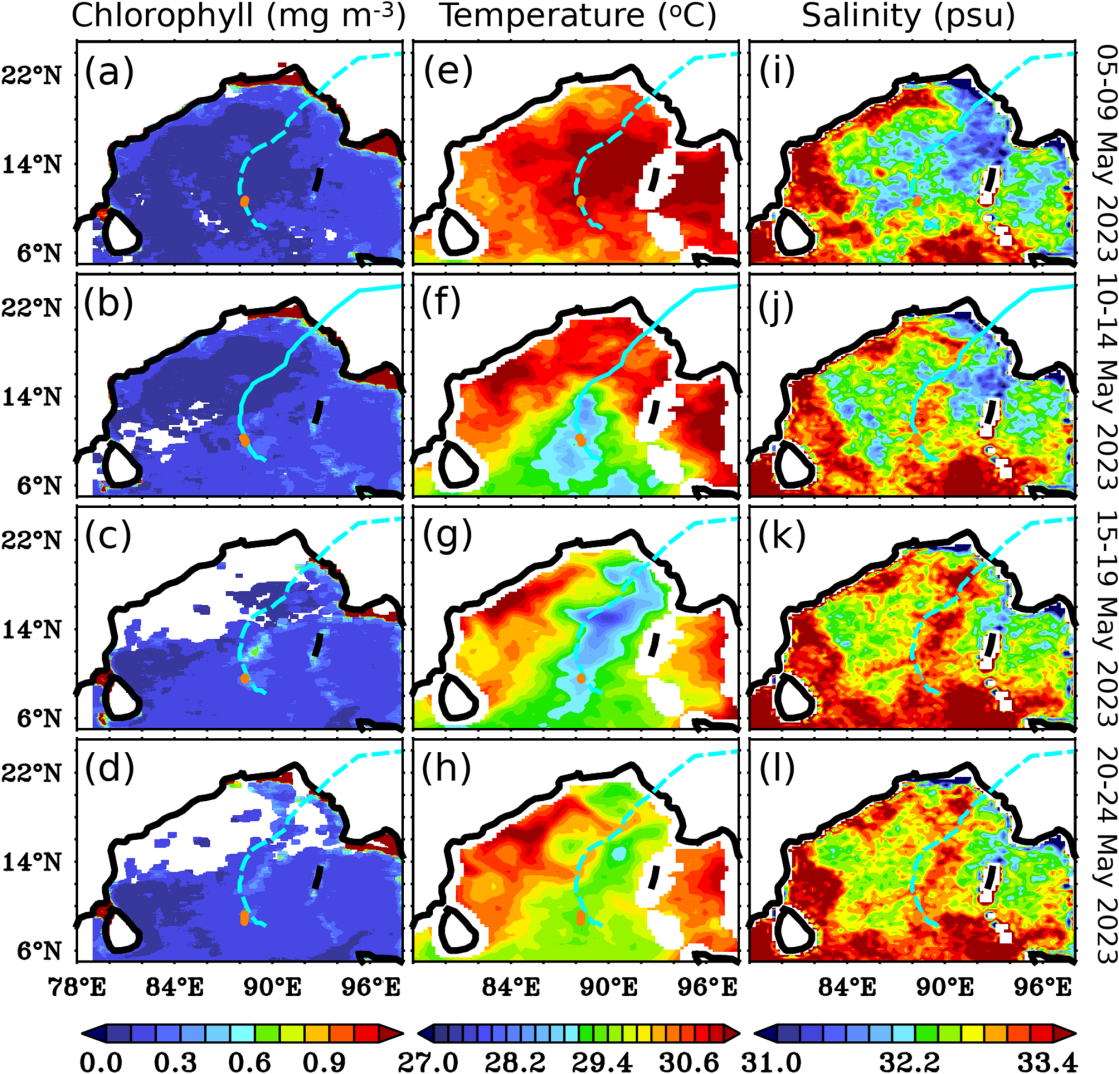
Five days composites of (**a**-**d**) Sea Surface chlorophyll (mg m^-3^) from GlobOcean color, (**e**-**h**) Sea Surface Temperature (°C) from MW-OI SST, and (i-l) Sea Surface Salinity (psu) from SMAP during TC *Mocha* ((**a**, **e** and **i**) 05–09 May 2023, (**b**, **f** and **j**) 10–14 May 2023, (**c**, **g**, and **k**) 15–19 May 2023 and (**d**, **h**, and **l**) 20–24 May 2023). The light blue line in the panels indicates TC tracks, and the orange circle indicates the glider’s location during that period. This figure was generated using PyFerret (v7.63).

The Sea Surface Salinity (SSS) from the glider also responded to TC, showing a mild increase of 0.9 psu, from 32.4 psu on 10 May 2023 to 33.3 psu on 12 May 2023 (Fig. 3b, and 3f). This salinity increase was clearly evident in the SMAP-SSS data, which showed a salting tendency along the TC’s track (Fig. 5i-l). Interestingly, unlike SST, the elevated SSS persisted for up to 10 days after the TC’s passage and remained noticeable until the end of May 2023 (Fig. 5i-l).

The temperature and salinity profiles from the glider showed a typical response in the temperature and salinity structure before (05 May) and after (14 May 2023) the passage of TC *Mocha* at the study location (Fig. 4). Before TC, the near-surface temperature observed from the glider was uniform at 30 °C, extending to a depth of 26 m. After the passage of the TC, the temperature in the upper 100 m of the water column was cooled due to the vertical mixing and strong upwelling induced by the TC. The depth of the 28.5^o^C isotherm, which was found to be at the depth of 45–50 m before TC, showed a sudden shallowing tendency after the passage of the TC, and reached upto the near-surface. The subsurface cooling persisted at the near-surface even upto 17 May, indicating the predominant role of the strong upwelling, which is also evident in the satellite data (Figs. 3e and 5e-h).

**Fig. 6:**
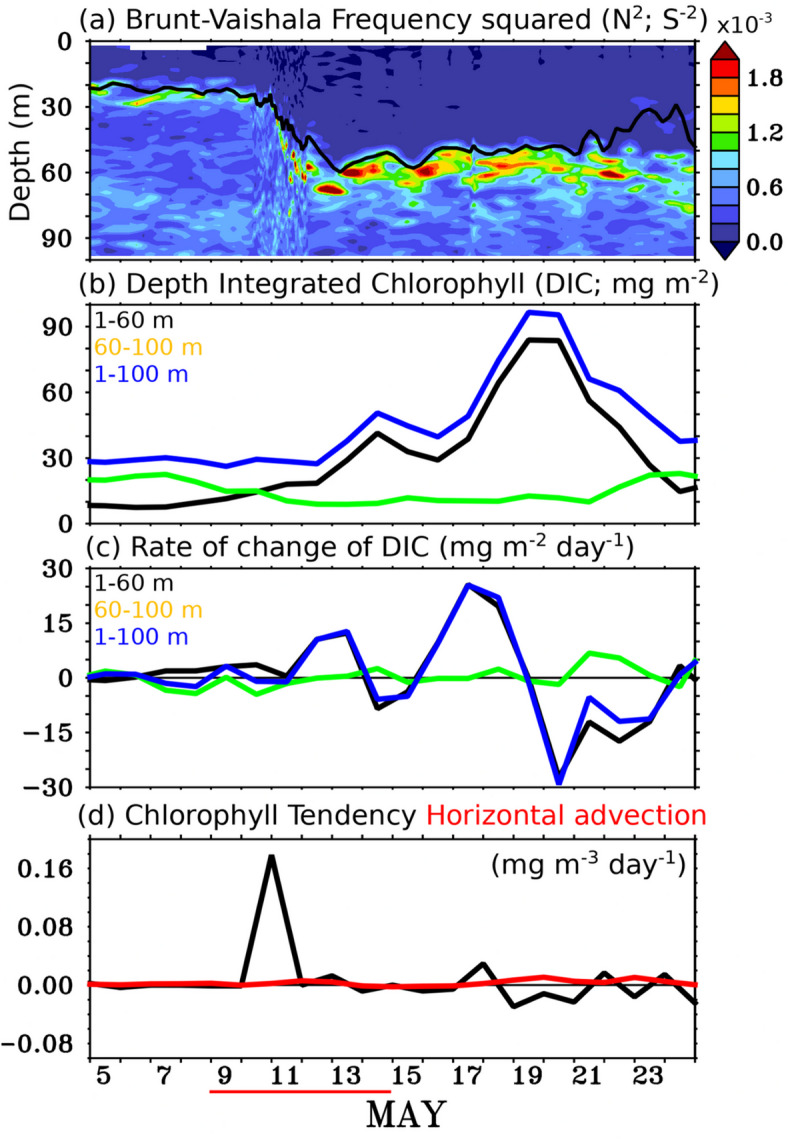
Temporal evolution (**a**) Brunt-Vaishala Frequency squared (N^2^; s^-2^) from glider measurements, (**b**) depth-integrated chlorophyll (DIC; mg m^-2^; 1–60 m is black line; 60–100 m is green line; 1–100 m is blue line) from glider measurements, (**c**) rate of change of DIC (mg m^-2^ day^-1^; 1–60 m is black line; 60–100 m is green line; 1–100 m is blue line) from glider measurements, and (**d**) sea surface chlorophyll tendency (black) and horizontal advection (red) (mg m^-3^ day^-1^) estimated from satellite data. The thick red line at the bottom of the figure indicates the TC *Mocha* period (09–15 May 2023). This figure was generated using PyFerret (v7.63).

The significant impact of a TC on ocean stratification and mixing can be understood using the Brunt-Vaisala frequency (N^2^). Prior to the TC’s passage, N^2^ was relatively low in the upper 20 m of the water column, while values above 20 m were highly positive, indicating a strongly stratified layer (Fig. 6a). However, as a result of TC-induced vertical mixing and turbulence from strong winds, the vertical stratification of the water column was disrupted, leading to the mixing of surface waters with deeper layers. Consequently, the water became less stratified, and the density gradient weakened, causing a decrease in N^2^ down to 50 m depth (Fig. 6a). With the reduced stratification, the water column became more unstable, allowing for increased vertical mixing. Eventually, the MLD and ILD deepened from 22 m to 57 m and 27 m to 60 m (on daily scales), respectively, due to this vertical mixing and upwelling induced by the TC (Fig. 3a and b and S4). The 23 °C isotherm (D23), which is considered as a proxy for the thermocline, also showed a shallowing tendency after the passage of the TC and shallowed up upto a depth of 77 m (after TC; 14 May) from a depth of 99 m (before TC; 09 May). After the TC passed, mixing gradually subsided, and the water column began to re-stratify, restoring more stable conditions.

### Quenching correction to the day fluorescence profiles from the glider

Before the correction, chlorophyll measurements from the glider showed stronger diurnal variability at the near-surface, with larger amplitude variations observed after the passage of the TC. This pattern was also evident at slightly deeper depths (Figure S1a and S2). Prior to the TC’s passage, the diurnal variability in near-surface chlorophyll was minimal, with amplitudes ranging from 0.02 to 0.04 mg m^-3^. However, after the TC, the amplitude of diurnal variability gradually increased to 1.2 mg m^-3^ (Figure S1a and S2). The near-surface chlorophyll concentration at night was nearly twice as high as those observed during the day. Statistical analysis revealed stronger diurnal variability in near-surface chlorophyll during the bloom period than during the non-bloom period. The standard deviations was 0.38 mg m^-3^ during the bloom period compared to 0.023 mg m^-3^ during the non-bloom period at night, and 0.18 mg m^-3^ (bloom) versus 0.029 mg m^-3^ (non-bloom) during the day. This observed diurnal variability in near-surface chlorophyll may be largely influenced by non-photochemical quenching of fluorescence (NPQ)^36,37^. During periods of high irradiance, the decrease in fluorescence quantum yield results in lower fluorescence levels than those observed at night^36–38^. This results in an inaccurate representation of chlorophyll concentration during the daytime, under-representing the true values and necessitating correction to understand the variability of phytoplankton biomass, especially during TCs. Thus, the daytime chlorophyll profiles were corrected for fluorescence quenching using the nighttime chlorophyll profiles, along with day and nighttime backscatter profiles from the glider, by following *Thomalla et al.* [2018]^38^. After performing NPQ-correction to the chlorophyll profiles, the dominant diurnal signal was suppressed, and the amplitude of the diurnal variability was significantly reduced at the near-surface (Figure S1b and S2). In the remaining section, we have used NPQ-corrected chlorophyll data from the glider for the analysis (Figure S1b).

### Variability of the upper ocean chlorophyll in response to TC *Mocha*

Similar to physical variables, biogeochemical variables measured by the glider also showed a substantial response following the passage of TC *Mocha*. Prior to the passage of the TC, the near-surface chlorophyll concentrations were very low (< 0.1 mg m^-3^), while the subsurface chlorophyll maxima (SCM; 0.4–1.3 mg m^-3^) persisted at depths of approximately 60–95 m (Fig. 3c). The persistence of the sub-surface chlorophyll maxima (SCM) at these depths is due to the net result of optimum availability of both light and nutrients, where the growth of phytoplankton is highly dependent on these two factors^31,33^. In response to the TC *Mocha*, the near-surface chlorophyll measured by the glider initially showed a mild enhancement, which gradually increased to 0.8 mg m^-3^ on 14 May and reached a peak of 1.7 mg m^-3^ on 19 May, approximately eight days after the TC passed over the study location (Fig. 3c and g, and 5a-d). The second near-surface chlorophyll peak (1.7 mg m^-3^), observed eight days after the passage of the TC *Mocha*, was significantly stronger than the first peak (0.8 mg m^-3^) that occurred immediately after the TC passed. This could be attributed to the large cloud cover during the first peak, which limited sunlight and affected photosynthesis at the study location (Fig. 2). The second near-surface chlorophyll peak was apparent from 18 to 21 May 2023, with enhanced chlorophyll concentrations extending down to a depth of ~55 m (Fig. 3c). Satellite-derived chlorophyll show a slight increase following the passage of the TC, but fail to capture the peak chlorophyll concentrations as observed in the glider measurements. This discrepancy may be attributed to limitations imposed by cloud cover, and the relatively coarse spatio-temporal resolution of the satellite data. Furthermore, the satellite-derived chlorophyll values represent domain-averaged estimates, which may not capture localized variations observed by the high-resolution glider measurements.

Chlorophyll profiles from the glider on 05 May 2023 (before the passage of TC) and 14 May 2023 (after the TC) illustrate the impact of the TC on chlorophyll distribution (Fig. 4i-l). Before the TC (05 May 2023), chlorophyll concentrations were low (0.15 mg m^-3^), and nearly uniform down to a depth of 60 m, with a SCM observed at approximately 62–95 m depth (Fig. 4i). After the TC passage, a mild increase in chlorophyll concentration (0.7-1.0 mg m^-3^) was observed down to 50 m on 14 May 2023, with the SCM absent (Fig. 4k-l). By 19 May 2023, higher chlorophyll concentrations (1.6-2.0 mg m^-3^) were observed extending to a depth of ~44 m.

The potential factors contributing to the near-surface chlorophyll enhancement following the passage of the TC are explored. These include the upward movement of subsurface chlorophyll-rich waters, the vertical transport of subsurface nutrients to the near-surface, and lateral redistribution through horizontal advection. These processes likely support the observed near-surface chlorophyll enhancement^31,33,34^ and are examined individually in the following paragraph.

The reduction in N^2^ signifies a weakening of the water column’s stability, which facilitates the upward movement of nutrients from deeper layers to the near-surface, potentially enhancing primary production in the upper ocean (Fig. 6a). Please note that the increase in nitrate concentration did not result in an immediate or rapid rise in chlorophyll concentration during the period of the TC. This is clearly evident from the depth-integrated chlorophyll measurements in the upper 100 m of the water column (Fig. 6b). The selection of depth-integrated chlorophyll up to 100 m is due to the lack of light beyond this depth, which leads to negligible chlorophyll variability below 100 m^34^.

Before the passage of the TC, the depth-integrated chlorophyll (up to 100 m) was 28 mg m^-2^, with the depth-integrated SCM lying between 60 and 100 m contributing 20 mg m^-2^, which accounted for approximately 71% of the total chlorophyll (Fig. 6b). On 14 May, chlorophyll concentration increased by about 81% to 51 mg m^-^² (Fig. 6b). This increase is likely due to the upward movement of the SCM to the near-surface, driven by TC-induced vertical mixing and upwelling, as evidenced by the deepening of the MLD (Fig. 3c). This analysis indicates that the SCM serves as a source of biomass for the rapid phytoplankton growth near the surface immediately after the TC. However, its contribution to the second chlorophyll peak observed at the near-surface during the bloom period is relatively weak (Fig. 6b and c). The depth-integrated chlorophyll (upto 100 m) peaked at 97 mg m^-2^ on 19 May, 8 days after the TC passed the study location. This represents nearly a threefold increase compared to the pre-TC depth-integrated chlorophyll and twice the concentration observed immediately after the TC (Fig. 6b). Please note that the SCM was not apparent on 12 May 2023, which may primarily be due to the vertical mixing caused by the TC and extensive cloud cover at the study location (Fig. 3c). Further, the presence of chlorophyll blooms near the surface might have prevented sunlight from penetrating to the subsurface layers, thereby hindering the formation of the SCM. As there was no substantial evidence of SCM contribution, we can speculate that the chlorophyll enhancement at the near-surface might be due to the new production, facilitated by optimal light and nutrient availability. This can be evident from the shallow tendency of the euphotic depth from 73 m (08 May 2023) to 29.6 m (19 May 2023) (Fig. 3c). The SCM, however, reappeared in the glider measurements starting from 22 May 2023 (Fig. 3c).

Consistent with earlier studies, the chlorophyll enhancement observed at the near-surface persisted even upto 10 days after the passage of the TC, and the recovery state of chlorophyll concentration to pre-TC periodic values was much slower than SST^33,34^ (Fig. 5). It was clearly evident from the satellite data along the track of the TC (Fig. 5c and d). Further, the abrupt increase in chlorophyll during the bloom period at the near-surface may indicate some contribution from the horizontal advection of chlorophyll into the study region. However, the spatial distribution of satellite chlorophyll does not show any evidence of chlorophyll enhancement, except along the track of the TC (Fig. 5a-d). Moreover, the estimation of horizontal advection using satellite-derived chlorophyll and surface current data indicates no significant horizontal transport of chlorophyll at the study location (Fig. 6d). Therefore, the role of horizontal advection in enhancing chlorophyll during this period can be ruled out, as horizontal transport merely redistributes existing pigment across spatial gradients and does not represent new primary production or carbon export associated with these processes.

### Variability of the upper ocean dissolved oxygen in response to TC *Mocha*

Before the passage of the TC *Mocha*, the near-surface dissolved oxygen (DO) concentrations were around 194–195 µM, extending down to ~20 m depth as observed by the glider. The subsurface high DO concentration (> 200 µM) was observed at depths of 25–60 m, and the oxycline was observed at depths of around 70–85 m.

The high-temporal resolution of glider measurements captured the variability in DO over the course of a day due to the impact of the TC. Initially, the DO concentration at the near-surface exhibited a mild reduction of approximately 2.8 µM, from 195.1 µM on 08 May 2023 to 192.3 µM on 09 May 2023, due to the influence of the TC. However, it increased by ~ 5.6 µM, reaching 197.9 µM on 11 May 2023, before experiencing a substantial drop of 12 µM, reaching a minimum of 185.9 µM on the same day (Fig. 3d and h). The observed reduction in the oxygen concentration at the near-surface layers could be due to the strong wind-induced vertical mixing and upwelling induced by the TC, which might lead to the intrusion of low-concentration DO subsurface water into the near-surface layers^33,34^. This is clearly evident from the contour of 175 µM, where the shallowing tendency was observed from 73 m (08 May 2023) to 42 m (12 May 2023) depth (Fig. 3d).

After the passage of the TC, the near-surface DO concentration showed a substantial increase from 18 to 24 May 2023, reaching its peak enhancement of 205 µM on 20 May 2023. This period of DO enhancement at the near-surface coincided with the period of maximum chlorophyll concentration enhancement at the near-surface (Fig. 3d and h). Please note that weaker wind speeds were observed during this period. Hence, the potential enhancement of DO in the near-surface layers due to strong winds induced by the TC, which may cause entrainment of oxygen from the overlying air, can be considered negligible^33,34,39^. Thus, the enhancement of oxygen at the near-surface layer must be a consequence of the enhancement of photosynthetically produced oxygen due to chlorophyll concentration enrichment in the near-surface layer in response to the TC *Mocha.* Further, the profiles of DO concentration on 05 May 2023 and 19 May 2023 showed the typical response to TC where enhanced oxygen concentration was observed and found to be uniform to 52 m depth (191–200 µM), which was almost consistent with the depth of high chlorophyll concentration (Fig. 4d).

### Diurnal variability of DO before and after the TC

It is worth pointing out that the DO measurements from the glider also showed strong diurnal variability at the near-surface after the passage of TC. Prior to the TC passage, the near-surface DO exhibited very small diurnal variability. Following the TC, the amplitude of DO variability increased, corresponding to the enhanced diurnal variability of chlorophyll(Fig. 3h). The standard deviation of near-surface oxygen concentration during daytime and nighttime was found to be 1.5 and 1.7 µM, respectively, during the non-bloom period. In contrast, during the bloom period, the standard deviation at the near-surface was 3.8 and 4.0 µM, respectively, during daytime and nighttime. This statistical comparison shows strong diurnal variability in the DO at the near-surface during the day and nighttime of the bloom period compared to the non-bloom period. During the bloom period, the DO at the near-surface had an enhanced oxygen concentration at night compared to that during the daytime, which corresponds to chlorophyll variability. Please note that the enhanced diurnal variability of DO observed during the bloom period was significantly lower (3.8-4 µM) than the accuracy of the optode sensor ( < ± 8 µM). However, the temporal evolution of DO concentration at the near-surface shows minimal diurnal variability before the TC (pre-TC), but exhibits significantly increased diurnal variability after the cyclone (post-TC), validating the reliability of measurements from the optode sensors.

The post-TC increase in diurnal variability of DO is attributed to the enhancement of chlorophyll concentrations at the near-surface in response to TC-induced vertical mixing and upwelling. This is because during the day, phytoplankton and other photosynthetic organisms in the ocean utilize sunlight to perform photosynthesis. This process produces oxygen as a byproduct, increasing DO in the water during daylight hours. At night, the photosynthesis process stops due to the lack of sunlight, but respiration continues. Respiration by both phytoplankton and other marine organisms consumes oxygen, leading to a decrease in DO levels at night. This photosynthesis–respiration cycle causes a diurnal fluctuation in oxygen concentrations, where oxygen levels are typically higher during the day (when photosynthesis is active) and lower at night (when respiration is the primary process).

### Evaluation of the model parameters with glider measurements in response to TC *Mocha*

**Fig. 7:**
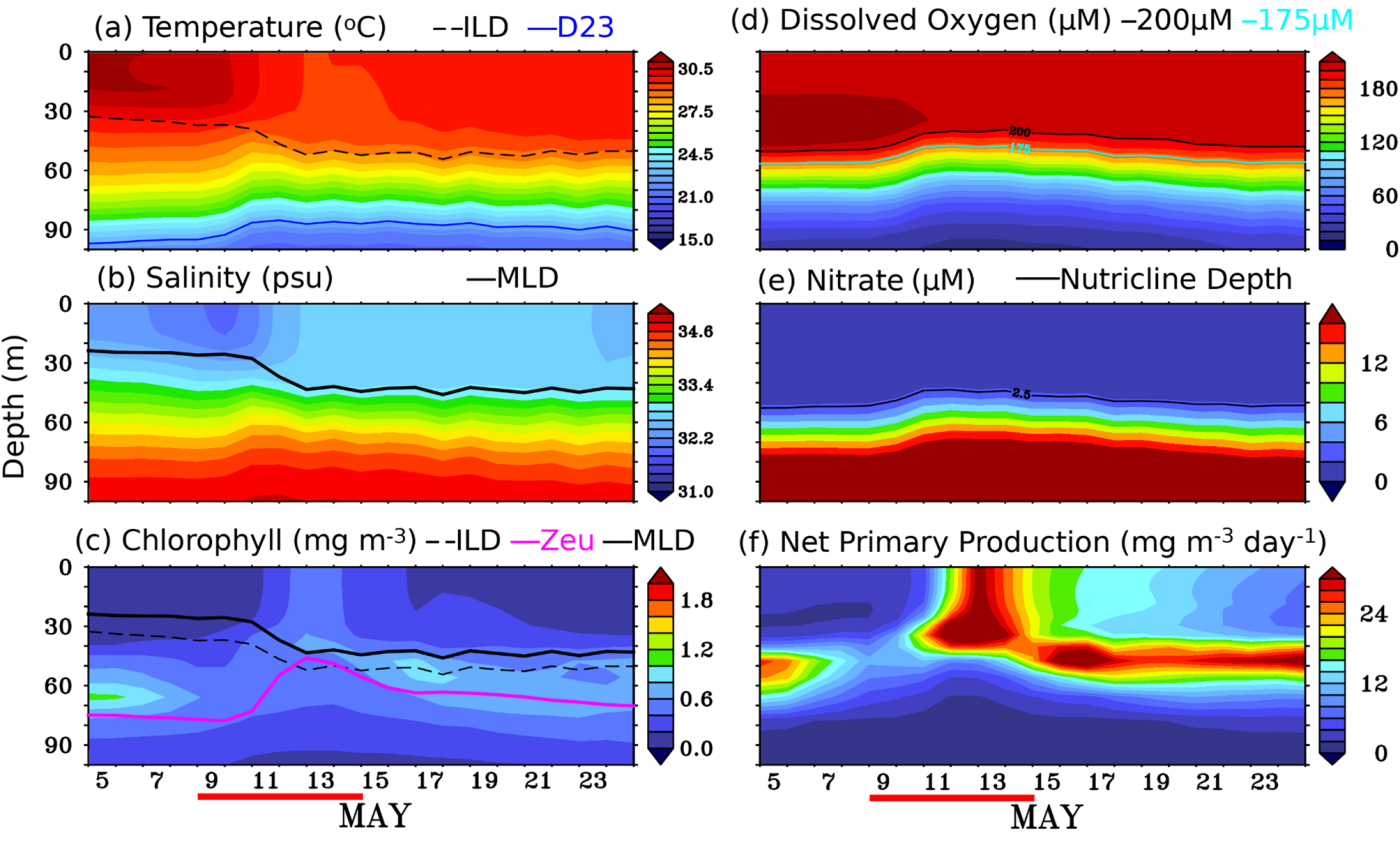
Depth-time section of (**a**) Temperature (°C), (**b**) Salinity, (**c**) Chlorophyll (mg m^-3^), (**d**) Dissolved Oxygen (µM), (**e**) Nitrate (µM), and (**f**) Net Primary Production (mg C m^-3^ day^-1^) from model data during 05–25 May 2023 on daily scales. In panel (**a**), thin dashed black and blue lines are ILD (m) and D23 isotherms; in panel (**b**), a thick black line is MLD (m). In panel (**c**), a thick pink line: euphotic depth, a thick black line is MLD (m), and a thin black dashed line: ILD (m). In panel (**d**), a thin lightblue (black) line: depth of 175 (200) µM dissolved oxygen (m). In panel (**e**), a thin black line is nutricline depth. All the parameters in the panels (**a**-**f**) from the model data were averaged over the box 8.4°N–10.8°N and 88°E–88.5°E. The thick red line at the bottom of the figure indicates the TC *Mocha* period (09–15 May 2023). This figure was generated using PyFerret (v7.63).

Figure 7 shows the depth-time section of the model simulated (a) Temperature (^o^C), (b) salinity (psu), Chlorophyll (mg m^-3^), (d) Dissolved oxygen (µM), (e) Nitrate (µM), (f) Net primary productivity (mg C m^-3^ day^-1^) on daily scales. Before the passage of the TC, the temperature and salinity structures from both the glider and the model exhibited similar characteristics, showing warm, low-salinity water at the near-surface and cold, high-salinity waters at the subsurface in the study region (Figs. 3a and b and 7a, and 7b). Both the MLD and ILD were found to be deeper in the model (23–24 m and 33–35 m depth) as compared to the glider (20–22 m and 27–29 m) (Figs. 3a and b and 7a, and 7b). Due to the impact of TC *Mocha*, the SST from the model dropped significantly; however, the rate of cooling in the model (~ 1.3 °C) was comparatively lower than that observed by the glider (~ 2.5 °C) (Fig. 3e and Figure S3a). It is important to note that the model temperature responded immediately during the passage of the TC, with cooling observed up to a depth of 20 m, in contrast to the observations (Figs. 3a and 4b, and S3a). However, the model might not have captured the full extent of vertical mixing and upwelling as observed in the glider measurements due to the impact of the TC (Figs. 3a, and 4b). The post-TC temperature profiles from the model indicated a slight warming than glider observations (Figures S3a and Fig. 4c-d). Although both the MLD and ILD deepen after the passage of the TC in the model (44 m and 52 m), they appear to be shallower compared to the glider measurements (57 m and 60 m) (Fig. 4a-d).

Similar to the post-TC temperature, the model’s response in salinity was weaker compared to the observations. The model showed higher salinity before the passage of the TC and a decrease in near-surface salinity during the TC, in contrast to the glider measurements (Figs. 3b and 4e-h, and S3b). Although the model indicated an increasing trend in near-surface salinity after the TC, the values remained lower than those observed.

The biogeochemical variables in the model also responded to the TC, but the response was weaker compared to the glider observations. Similar to glider measurements, the model estimated chlorophyll at the near-surface, found to be very small. The SCM, observed at depths of 60–95 m in the observations, is located between 45 and 90 m in the model prior to the passage of the TC (Figs. 3c and 4i, and 7c). Due to the impact of the TC, the model-estimated chlorophyll at the near-surface showed an immediate increase on 12 May 2023 (0.31 mg m^-^³), reaching a peak of 0.54 mg m^-^³ on 13 May (Figure S3). Afterward, it began to decrease, returning to pre-cyclonic levels shortly after the cyclone passed. It is worth noting that while the first chlorophyll peak occurred immediately after the cyclone’s passage in the model (0.3 mg m^-^³ on 12 May), it was slightly delayed and of higher magnitude in the observations (0.8 mg m^-^³ on 14 May) (Figs. 3c and g and 7c, and S3c). Furthermore, the second chlorophyll peak, observed in the glider measurements eight days after the cyclone passed the study location, was not noticed in the model-estimated chlorophyll (Figure S3c). The peak magnitude of chlorophyll observed from glider measurements was three times higher than the peak observed in the model-estimated chlorophyll due to post-TC impact. The SCM, which was apparent twelve days after the passage of the TC in the glider measurements, appeared immediately after the cyclone’s passage in the model-estimated chlorophyll.

The model-estimated DO (200 µM) was slightly higher than the observations (194–195 µM) at the near-surface throughout the study period (Figs. 3d, S3d, and 7d). Furthermore, the subsurface high DO was noticed between 20 and 50 m in the model, which is slightly shallower than the glider-measured DO (25–60 m) during the pre-TC period. Similarly, the oxycline depth in the model was also found to be shallower than glider measurements before and after the TC passage (Figs. 3d and 4m-p, and 7d). The model-estimated DO showed a mild response, increasing by 4 µM (from 200 µM to 204 µM) in response to the TC, but it remained consistently higher than the observations (Figs. 3h and S3d).

As mentioned in the earlier section on upper ocean variability of chlorophyll in response to the TC, the increase in chlorophyll at the near-surface observed eight days after the TC passage may be attributed to new production. But, we do not have in-situ nitrate measurements during this TC period. However, previous studies have shown a strong correlation between ILD and nutricline depth, indicating that the ILD can serve as a reliable proxy for nutricline depth in these observations^33,34^. The model-simulated nitrate showed a mild increase at the near-surface level (~ 0.12 µM) (Fig. 7e). The nutricline depth, estimated from model nitrate data, showed a slight shallowing trend, reaching its shallowest point on 12 May 2023 (from 52 m to 43.5 m) due to the TC’s impact (Fig. 7e). Meanwhile, the ILD, as observed and modeled, exhibited a deepening trend due to the TC, bringing it close to the nutricline depth and showing strong temporal correspondence (Figure S4). This trend coincided with the peak of the chlorophyll bloom at the near-surface on 19 May (Figure S4). These analyses suggest that the upward flux of subsurface chlorophyll-rich waters and the availability of subsurface nutrients at the near-surface, driven by vertical mixing and upwelling during the TC-induced turbulence, likely contributed to new production, explaining the enhanced chlorophyll concentrations observed during the post-TC period^31,33,34^. Please note that the study area is situated well away from the river mouth, ensuring that the near-surface measurements were unaffected by suspended inorganic sediments. Moreover, the model-simulated primary productivity (PP) also showed an enhancement (29 mg C m^-3^ day^-1^) from 3 mg C m^-3^ day^-1^ (09 May) to 32 mg C m^-3^ day^-1^ (13 May) (Fig. 7f). The model-simulated PP showed immediate response to impact of the TC, which is consistent with the temporal evolution of model-simulated chlorophyll (Fig. 7c and f).

## Summary and Conclusion

Although significant progress has been made in ocean observation technologies over the past few decades, understanding air-sea interactions and upper ocean processes, particularly during tropical cyclones (TC), remains limited in the Bay of Bengal (BoB). Furthermore, high-resolution measurements in the upper ocean during extreme weather events are crucial for assessing the performance and capabilities of coupled biophysical ocean models, yet such data is poorly documented across the global ocean. Thus, the autonomous underwater vehicles, such as gliders, which can be operated from shore, offer a valuable solution to bridge this gap.

The deep-sea glider deployed as a part of Deep Ocean Mission (DOM) by Indian National Centre for Ocean Information Services (INCOIS), Ministry of Earth Sciences (MoES), had an opportunity to cross the track of TC *Mocha* (9–15 May 2023) on 10–11 May 2023 in the BoB and provided unprecedented high temporal and vertical resolution of upper ocean temperature, salinity, chlorophyll a, optical backscatter, and dissolved oxygen measurements. Thus, the first deep-sea glider measurements before and after the passage of the TC *Mocha* during the pre-monsoon (secondary TC) season provides a unique opportunity to examine the observed variability in the physical and biogeochemical responses of the upper ocean to the TC. These observations also helped to evaluate the performance and capabilities of the model in simulating key parameters.

Due to the impact of the TC *Mocha*, both atmospheric and oceanic variables showed substantial responses in the study region. The atmospheric variables and fluxes exhibited a strong response from May 10 to 12, 2023, at the study location. The OLR showed a strong reduction (119 W m^-2^), and wind stress exhibited a maximum enhancement (0.59 N m^-2^), resulting in the Ekman pumping reaching a maximum of 6 m day^-1^. In response to the TC, a maximum cooling of -402 W m^-2^ was observed in the net surface heat flux (Q_net_) on May 11, 2023 with maximum contribution of cooling from latent heat flux (Q_lat_; -375 W m^-2^).

Our analysis from glider shows a significant reduction in the SST (~ 2.5 °C) and maximum enhancement in the SSS (~ 0.9 psu), chlorophyll concentration (1.6 mg m^-3^), and dissolved oxygen (10 µM) measurements at the near-surface in response to the TC *Mocha*. High-temporal resolution glider measurements revealed diurnal variability in SST before and after the passage of the TC. Notably, the diurnal variation in SST was absent during the TC period. Following the passage of the TC, vertical mixing and strong upwelling cooled the temperature in the upper 100 m of the water column, shallowed the 28.5 °C isotherm from 50 m to near the surface, and deepened both the MLD and ILD. This is clearly reflected in N², where the vertical stratification of the water column was disrupted, causing the mixing of surface waters with deeper layers due to TC-induced vertical mixing and upwelling.

It is worth pointing out from the chlorophyll measurements that the second near-surface chlorophyll peak (1.7 mg m^-3^) observed eight days after the TC passage was significantly stronger than the first chlorophyll peak (0.8 mg m^-3^) that occurred immediately after the passage of the TC. This might be due to the large cloud cover present during the first chlorophyll peak period at the near-surface. Although satellite-derived chlorophyll shows a slight increase after the TC passage but fails to capture the peak concentrations as seen in high resolution glider measurements. This discrepancy is likely due to cloud cover and the coarse spatio-temporal resolution of the satellite data. Analysis of depth-integrated chlorophyll (up to 100 m) from glider measurements, before and after the TC passage, suggests that the SCM acts as a source of biomass for rapid phytoplankton growth near the surface during the initial chlorophyll peak following the TC. This is likely due to the upward flux of subsurface chlorophyll-rich waters to the near-surface, driven by TC-induced vertical mixing and upwelling. The depth-integrated chlorophyll showed nearly a threefold increase on 19 May 2023 compared to the pre-TC depth-integrated chlorophyll and twice the concentration observed immediately after the TC. As there is no evidence of SCM during this period, the role of SCM contribution to the latter chlorophyll enhancement is likely negligible. Hence, we can speculate that the latter chlorophyll enhancement in near-surface may be attributed to new production, driven by optimal light and nutrient availability.

High-temporal resolution of glider measurements captured diurnal variability in dissolved oxygen (DO) influenced by the passage of the TC *Mocha*. Due to the impact of the TC, near-surface DO concentrations experienced a substantial reduction (12 µM), likely caused by strong wind-induced vertical mixing and upwelling, which may have led to the intrusion of low-DO subsurface water into the near-surface layers. Following this, DO concentrations at the near-surface increased by 10 µM, coinciding with the period of enhanced chlorophyll concentrations. During this DO enhancement, wind speeds were weak, which ruled out the role of wind-driven oxygen entrainment from the overlying air. Therefore, the increase in near-surface oxygen must be attributed to photosynthetically produced oxygen, resulting from the chlorophyll enrichment in the near-surface layer in response to the TC *Mocha*.

Statistical comparison reveals stronger diurnal variability in near-surface DO during the bloom period compared to the non-bloom period, corresponding to the increased diurnal variability in chlorophyll due to the TC impact. During the bloom period, near-surface DO concentrations were higher at night than during the day. This diurnal variability in DO is likely attributed to the photosynthesis–respiration cycle, which causes fluctuations in oxygen concentrations. Consistent with earlier studies, the chlorophyll concentration and DO measurements at the near-surface from the glider showed a maximum peak 8–9 days after the passage of the TC.

The evaluation of model-simulated parameters with glider measurements reveals weaker response to TC in the model as compared to glider measurements. The model-simulated SST exhibited a reduced cooling rate (~ 1.3 °C) compared to glider measurements (~ 2.5 °C). Post-TC temperature profiles from the model showed slight warming, in contrast to the glider observations. This discrepancy suggests that the model may not have fully captured the extent of vertical mixing and upwelling induced by the TC, as observed in the glider data. Although the model indicated an increasing trend in near-surface salinity after the TC, the values remained lower than those observed. Further, the model estimated an immediate increase in near-surface chlorophyll, peaking at 0.54 mg m^-3^ on 13 May, before returning to pre-cyclonic levels immediately. While the model captured the first chlorophyll peak, it was delayed and higher in the observations. Additionally, the model did not capture the second near-surface chlorophyll peak as observed in the glider measurements eight days after the TC passed over the study location. The model-estimated DO showed a mild response, increasing by 4 µM (from 200 µM to 204 µM) in response to the TC, but it remained consistently higher than the observations.

While this study highlights the importance of documenting the variability of upper ocean physical and biogeochemical variables using gliders in response to the TC, and evaluating the model’s performance, it has limitations regarding the measurement of turbulence and eddy diffusivity. However, the addition of a micro-rider on the glider could provide further insights into these aspects. Hence, accurate representation of these parameters in coupled biogeochemical models is crucial for realistic simulations of marine ecosystems and their responses to environmental changes.

## Data and Methods

### The life cycle of TC *Mocha*

The India Meteorological Department first reported the Depression (D) on 9 May 2023 at 1200 UTC, which intensified into a Deep Depression (DD) within 6 h on 10 May 2023 at 0000 UTC (Fig. 1). Then, TC intensified into a Cyclonic Storm (CS) and a Severe Cyclonic Storm (SCS) on 11 May 2023 at 0000 and 1200 h, respectively. Further, the TC intensified into a Very Severe Cyclonic Storm (VSCS) and Extremely Severe Cyclonic Storm (ESCS) on 12 May 2023 at 0000 and 1500 h, respectively (Fig. 1). Later, TC crossed the North Myanmar and Southeast Bangladesh coast on 14 May 2023 between 0700 and 0900 UTC and caused severe damage to lives and properties. The system weakened into a well-marked low-pressure area on 15 May 2023 over Northeast Myanmar and its neighbourhood.

### Glider data

The deep-sea gliders (SGs) from M/s. Teledyne Webb Research (TWR; Slocum G3 Glider) is a buoyancy-driven AUV, observing the physical and biogeochemical variables upto 1000 m depth in the water column with long range and duration^40–42^. The horizontal and vertical velocities of gliders in the water are 0.27 m s^-1^ and 0.09 m s^-1^, respectively. The deep-sea glider took very high vertical measurements (every meter) of Conductivity-Temperature-Depth (CTD; pumped) from Sea-Bird, Chlorophyll-a at 470/695 nm (range: 0–50 µg/l Chl-a), Backscatter at 700 nm (0–3 m^-1^ cp.), and CDOM at 370/460 nm (0-375 ppb) (FLBBCD) from WET LABS ECO and DO from Aanderaa optode. The measurement range of the Aanderaa optode is 0-500 µM with an accuracy of < ± 8 µM (Table 1). Initially, the glider was performing a north-to-south transect in the BoB, which was initially configured to measure only downward profiling up to 1000 m depth with surfacing every 5–6 h. During TC, the glider was reconfigured to obtain measurements at very high temporal (surfacing every 30–40 min) and vertical resolution (upto 200 m depth at 1 m intervals), from 10 to 12 May 2023. The glider data are quality controlled following *Troupin et al.* [2015]^43^.

**Table 1:** Variables measured by the Glider, along with range, accuracy, and study period.

S. No.	Sensor	Parameters	Range	Accuracy
**1.**	Pumped CTD	Temperature	0–35 °C	± 0.002 °C
		Salinity	20–40 PSU	± 0.005 PSU
		Pressure	0-1000 decibar	± 3 decibar
	Dissolved Oxygen (optical)		0-500 µmol/kg	± 8 µmol/kg
	Chlorophyll (optical)		0–25 µg/I	± 0.2 µg/l
	Backscatter at 700 nm (optical)		0–3 m^-1^	0.003 m^-1^
	Photosynthetically Available Radiation (PAR)		400–700 nm	± 2 nm
	Coloured Dissolved Organic Matter (CDOM) (optical)		0-375 ppb	0.3 ppb
**2.**	Study Period		05–25 May 2023	
**3.**	Tropical Cyclone *Mocha* Period		09–15 May 2023	

The fluorescence and backscatter data have been processed through several steps, including cleaning, despiking, and smoothing. The cleaning of data identified bad profiles using the IQR1.5 rule (interquartile range [IQR]), i.e., elements more than 1.5 interquartile ranges above the upper quartile (75th percentile) or below the lower quartile (25th percentile). The spikes in the data were removed, and the missing gaps were linearly interpolated to facilitate the analysis. Here, we have considered glider measurements at 3 m as the surface.

### Other datasets

In addition to deep-sea glider data, we have utilized various types of satellite data in this study. The 8-day running-average Soil Moisture Active Passive (SMAP) sea surface salinity (SSS) at 25-km resolution^44^, daily merged satellite product of chlorophyll-*a* at spatial resolution of 4 km from GlobColour^45,46^, daily (non-interpolated) Outgoing Longwave Radiation (OLR) from NOAA^47^, the Advanced Scatterometer (ASCAT) ocean surface wind data^48^ with a spatial resolution of 0.25° and ERA5 hourly data of radiative and turbulent heat fluxes from Copernicus Climate Change Service (C3S), Microwave Optimum Interpolation Sea Surface Temperature (MW-OI SST) with 0.25^°^ (~ 25 km) grid spacing from Remote sensing systems^49^ and three-hourly best TC tracks of TC *Mocha* from the India Meteorological Department (IMD) are used in this study. The Ekman pumping velocity (positive for upwelling and negative for downwelling) at the base of the Ekman layer is estimated using the expression of *Fischer* [1997]^50^ and *Girishkumar et al.* [2013b]^51^. The MLD is defined as the depth at which the *σ*_*t*_ (sigma-t) exceeds that at the surface, with the increase in *σ*_*t*_ caused by a 0.8^°^C change in temperature^22,8,34^, and the isothermal layer depth (ILD) is defined as the depth where the temperature is 1^°^C lower than SST.

### Model datasets

The temperature and salinity data obtained from the GLORYS12V1 product is the CMEMS global ocean eddy-resolving (1/12° horizontal resolution, 50 vertical levels) reanalysis covering the altimetry (1993 onward). The model component is the NEMO platform driven at surface by ECMWF ERA-Interim then ERA5 reanalyses for recent years. The chlorophyll, dissolved oxygen, nitrate, and primary productivity were obtained from the Operational Mercator Ocean biogeochemical global ocean analysis and forecast system at 1/4 degree. The global ocean output files are displayed with a 1/4 degree horizontal resolution with regular longitude/latitude equirectangular projection. 50 vertical levels are ranging from 0 to 5700 m.

## Supplementary Information

Below is the link to the electronic supplementary material.

Supplementary Material 1

## Data Availability

All the datasets analyzed in the current study are available in the data repositories as mentioned here, cited in the Data and Methods section, and acknowledgments. Most datasets used in the present study are open-source, while a few are available upon reasonable request from INCOIS ( [https://www.incois.gov.in/site/datarequisitionform.jsp]) and are described below. ASCAT data are produced by Remote Sensing Systems and sponsored by the NASA Ocean Vector Winds Science Team. The data can be downloaded from the INCOIS website by selecting ASCAT data products from the dataset at this link: [https://las.incois.gov.in/]. SMAP salinity data are produced by remote sensing systems and sponsored by the NASA Ocean Salinity Science Team, and the data are available by choosing the HTTPS Salinity data from the Download data menu option at the following link: [www.remss.com](http:/www.remss.com). ACRI-ST, France, developed, validated, and distributed the GlobColour data, which is available from the following link: https://hermes.acri.fr/index.php. The glider data is available from the INCOIS website for research purposes, and it can be downloaded upon request from the following link: [https://www.incois.gov.in/site/datarequisitionform.jsp]. Microwave OI-SST data are produced by Remote Sensing Systems and sponsored by the National Oceanographic Partnership Program (NOPP) and the NASA Earth Science Physical Oceanography Program, and the data are available by choosing the HTTPS OI SSTs from the Download data menu option at the following link: [www.remss.com]. NOAA Daily (non-interpolated) Outgoing Longwave Radiation (OLR) data provided by the NOAA PSL, Boulder, Colorado, USA, from their website at https://psl.noaa.gov/data/gridded/data.uninterp_OLR.html. Three-hourly best TC tracks of TC *Mocha* were obtained from the Regional Specialised Meteorological Centre (RSMC), India Meteorological Department (IMD), India ([https://rsmcnewdelhi.imd.gov.in/download.php? path=uploads/report/33/33\_31869f\_BestTrack\_2023%20\_ACR\_AMR\_Modified.pdf]). This study has been conducted using E.U. Copernicus Marine Service Information; [https://doi.org/10.48670/moi-00021]; [https://doi.org/10.48670/moi-00015].
