# Supplementary material for: Observed physical and biogeochemical variability due to tropical cyclone Mocha using glider observations in the Bay of Bengal
Source: Sci Rep. 2026 Apr 21;16:13009. doi: 10.1038/s41598-026-43528-2 (PMC13099991; doi:10.1038/s41598-026-43528-2)
Supplement: Supplementary file 1 — Supplementary Material 1 [file 41598_2026_43528_MOESM1_ESM.docx]

***(Scientific Reports)***

**Supporting Information for**

**Observed physical and biogeochemical variability due to Tropical Cyclone Mocha using glider observations in the Bay of Bengal**

V. P. Thangaprakash^1*^, N. Sureshkumar^1^, K. Siva Srinivas^1^, A. Chandramouli^1^, Sai Theagarajan^1^, Y. Rajasekhar^1^, Virendra Kumar^1^, and M. Ramesh Kumar^1^

^1^Indian National Centre for Ocean Information Services, Hyderabad, India.

^*^Corresponding author address: V. P. Thangaprakash, Indian National Centre for Ocean Information Services (INCOIS), Hyderabad-500090, INDIA. Email: [thangaprakash.vp@incois.gov.in](mailto:thangaprakash.vp@incois.gov.in), Phone: +91-40-23886168, Fax: +91-40-23892910.


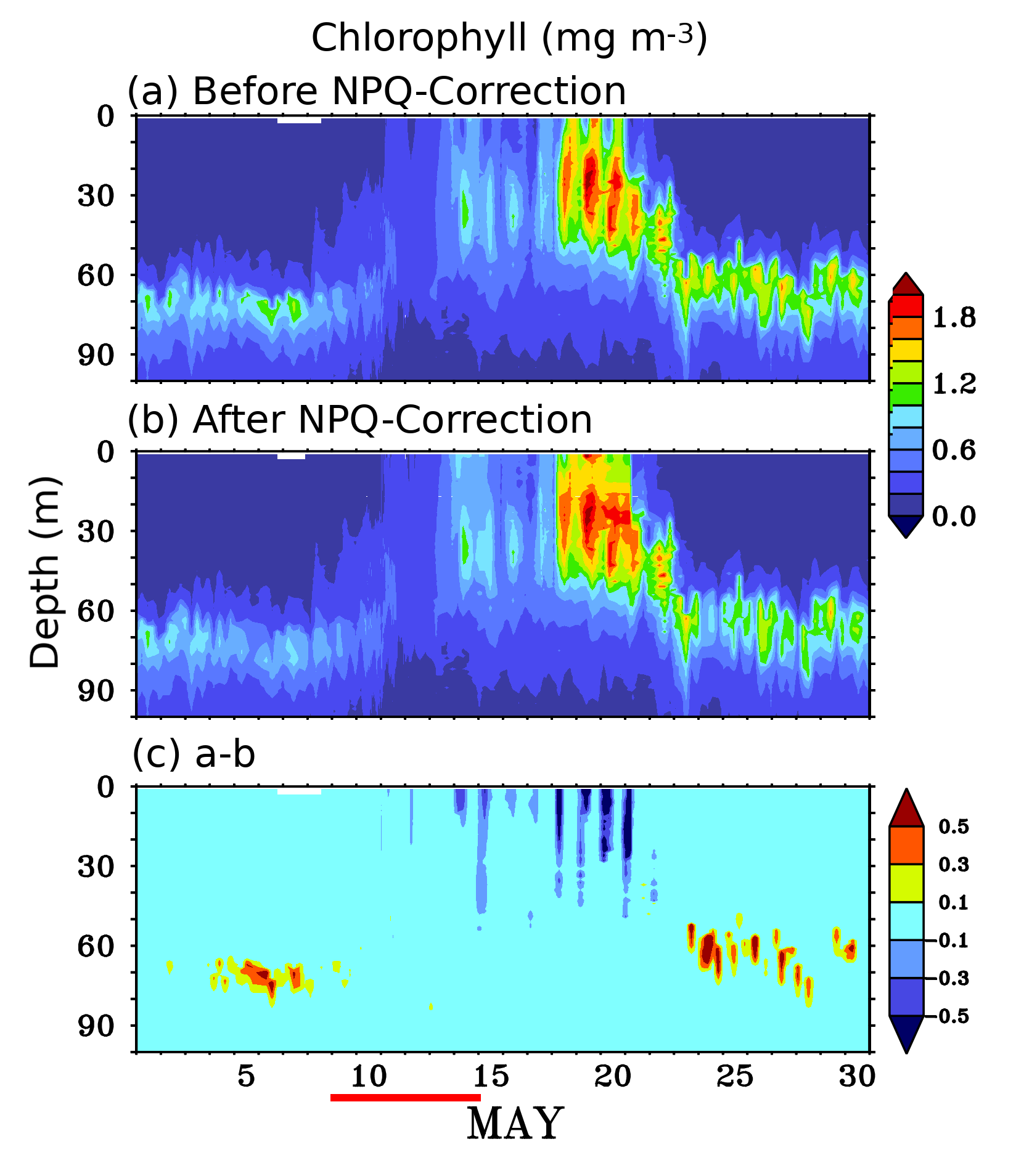


Figure S1. Time-depth section of chlorophyll (mg m^-3^) (a) before NPQ-correction and (b) after NPQ-correction (c) difference between before and after NPQ-corrected chlorophyll from the glider measurements. The thick red line at the bottom of the figure indicates the TC *Mocha* period (09–15 May 2023).


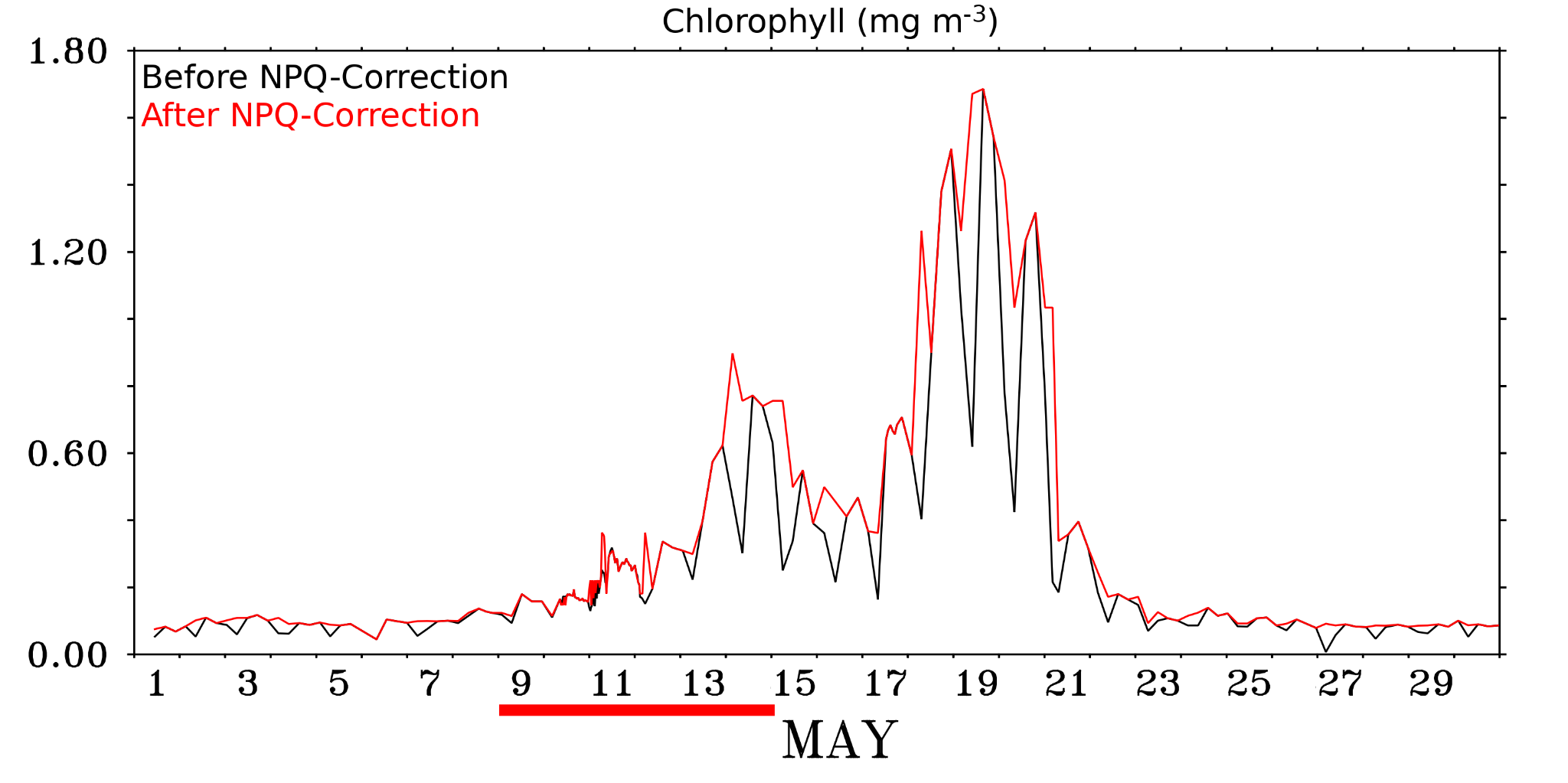


Figure S2. Temporal evolution of chlorophyll (mg m^-3^) before (black line) and after (red line) NPQ-correction from the glider measurements (3 m depth). The thick red line at the bottom of the figure indicates the TC *Mocha* period (09–15 May 2023).


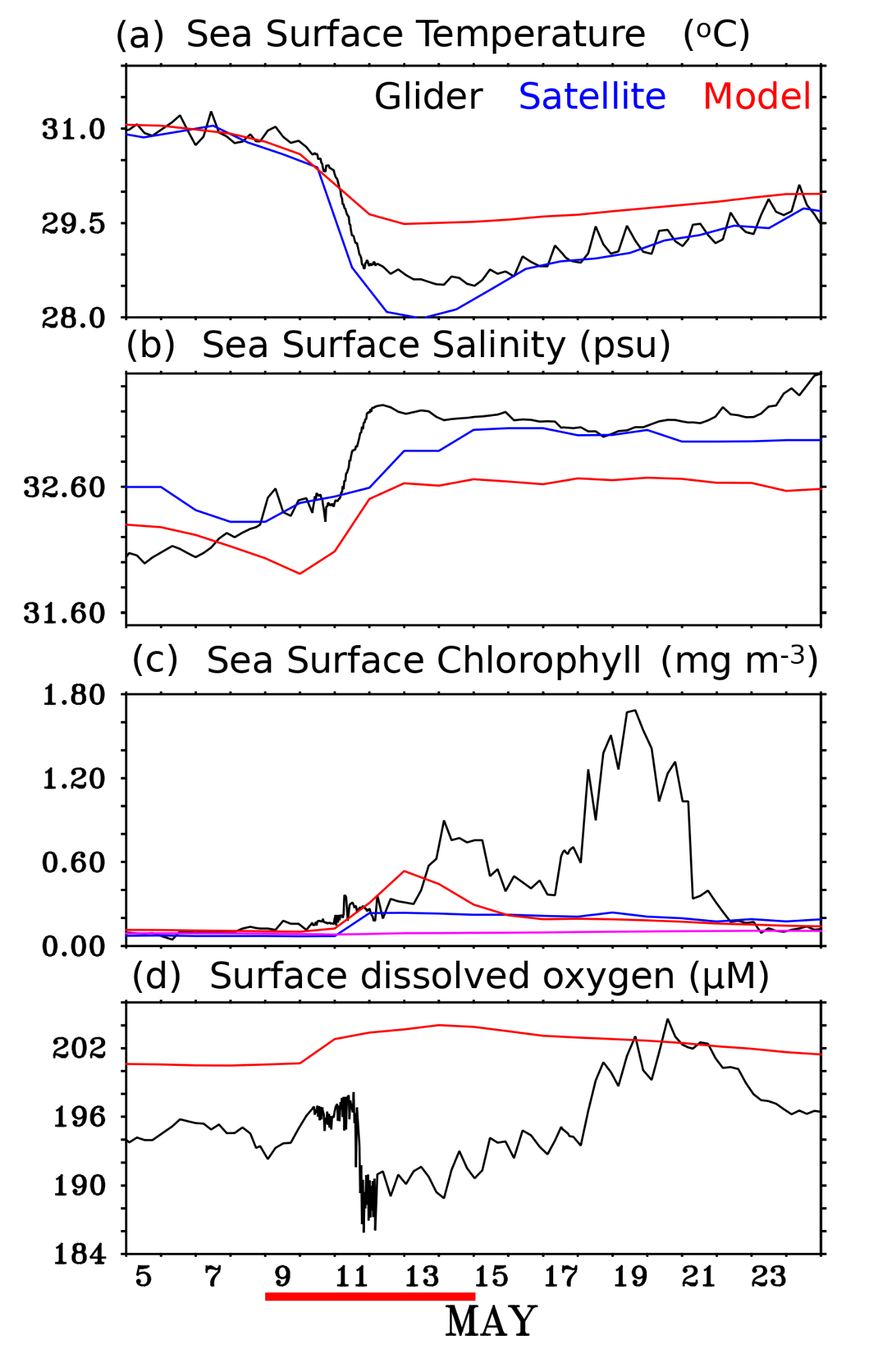


*Figure S3.* Temporal evolution of (a) sea surface temperature (°C), (b) sea surface salinity, (c) sea surface chlorophyll (mg m^-3^), and (d) sea surface dissolved oxygen (µM) from the glider (black), model (red), and satellite (blue) measurements. The blue line in panels (a), (b), and (c) represents data from satellite sources: MW-OI SST, SMAP, and GlobOcean color. The satellite and model data were averaged over the box 8.4°N–10.8°N and 88°E–88.5°E. The thick red line at the bottom of the figure indicates the TC *Mocha* period (09–15 May 2023).


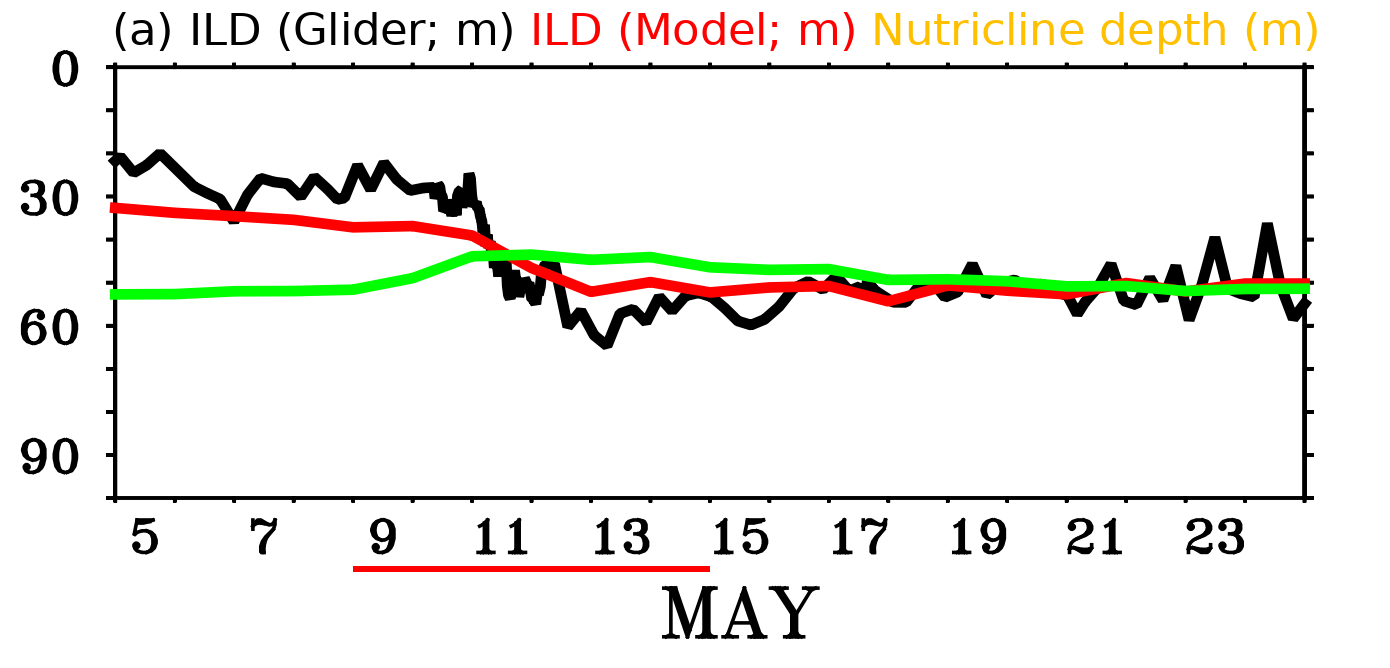


Figure S4. ILD from Glider (black) and Model (red), along with nutricline depth (green) from 05-25 May 2023.
